# Performance of Hepatitis C Virus (HCV) Core Antigen Assay in the Diagnosis of Recently Acquired HCV Infection among High-Risk Populations

**DOI:** 10.1128/spectrum.00345-22

**Published:** 2022-05-17

**Authors:** Hsin-Yun Sun, Wang-Da Liu, Chih-Wen Wang, Yu-Ju Wei, Kuan-Yin Lin, Yu-Shan Huang, Li-Hsin Su, Yi-Ting Chen, Wen-Chun Liu, Yi-Chin Su, Yea-Wen Chen, Yu-Chung Chuang, Po-Liang Lu, Chien-Ching Hung, Ming-Lung Yu

**Affiliations:** a Department of Internal Medicine, National Taiwan University Hospital and National Taiwan University College of Medicine, Taipei, Taiwan; b Department of Medicine, National Taiwan University Cancer Center, Taipei, Taiwan; c Hepatobiliary Division, Department of Internal Medicine, Kaohsiung Medical University Hospital, Kaohsiung Medical University, Kaohsiung, Taiwan; d Department of Internal Medicine, Kaohsiung Medical University Hospital and College of Medicine, Kaohsiung Medical University, Kaohsiung, Taiwan; e Department of Tropical Medicine and Parasitology, National Taiwan University College of Medicine, Taipei, Taiwan; f Department of Medical Research, China Medical University Hospital, Taichung, Taiwan; g China Medical University, Taichung, Taiwan; h School of Medicine and Hepatitis Research Center, College of Medicine, Kaohsiung Medical University, Kaohsiung, Taiwan; i Institute of Biomedical Sciences, National Sun Yat-Sen University, Kaohsiung, Taiwan; Quest Diagnostics Nichols Institute

**Keywords:** men who have sex with men, sexually transmitted infection, viral hepatitis, preexposure prophylaxis, HCV viremia, pooled-plasma HCV RNA testing, sensitivity, specificity, positive predictive value, negative predictive value, HCV core antigen assay

## Abstract

How the hepatitis C virus (HCV) core antigen (HCVcAg) assay performs in detecting recently acquired HCV infection among people living with HIV (PLWH) and HIV-negative men who have sex with men (MSM) is rarely assessed in the Asia-Pacific region. High-risk participants, including PLWH with sexually transmitted infections (STIs), HCV clearance by antivirals or spontaneously, or elevated aminotransferases, HIV-negative MSM with STIs or on HIV preexposure prophylaxis, and low-risk PLWH were enrolled. Blood samples were subjected to 3-stage pooled-plasma HCV RNA testing every 3 to 6 months until detection of HCV viremia or completion of the 1-year follow-up. The samples at enrollment and all of the archived samples preceding the detection of HCV RNA during follow-up were tested for HCVcAg. During June 2019 and February 2021, 1,639 blood samples from 744 high-risk and 727 low-risk PLWH and 86 HIV-negative participants were tested for both HCV RNA and HCVcAg. Of 62 samples positive for HCV RNA, 54 (87.1%) were positive for HCVcAg. Of 1,577 samples negative for HCV RNA, 1,568 (99.4%) were negative for HCVcAg. The mean HCV RNA load of the 8 individual samples positive for HCV RNA but negative for HCVcAg was 3.2 (range, 2.5 to 3.9) log_10_ IU/mL, and that of the remaining 54 samples with concordant results was 6.2 (range, 1.3 to 8.5) log_10_ IU/mL. The positive predictive value (PPV) and negative predictive value (NPV) of HCVcAg were 85.7% and 99.5%, respectively. In at-risk populations, HCVcAg has a high specificity and NPV but lower sensitivity and PPV, particularly in individuals with low HCV RNA loads.

**IMPORTANCE** The HCV core antigen assay has a high specificity of 99.4% and negative predictive value of 99.5% but a lower sensitivity of 87.1% and positive predictive value of 85.7% in the diagnosis of recently acquired HCV infection in high-risk populations. Our findings are informative for many countries confronted with limited resources to timely identify acute HCV infections and provide effective direct-acting antivirals to halt onward transmission.

## INTRODUCTION

With the advances of direct-acting antivirals (DAAs) against hepatitis C virus (HCV) (1, 2), timely diagnosis of HCV infection and linkage to HCV treatment can never be overemphasized to curb its onward transmission and avoid subsequent hepatic and extrahepatic complications. The populations at higher risk for repeat infections who contribute to most HCV infections, such as sexually active men who have sex with men (MSM) and people who inject drugs (3–6), will benefit most from this treatment-as-prevention strategy. In real-world settings, this strategy has effectively led to declines in HCV incidence and prevalence among people living with HIV (PLWH) in several developed countries (7–10).

The World Health Organization (WHO) advocates global hepatitis C elimination by 2030 (11). To effectively implement the treatment-as-prevention program for HCV elimination, timely identification of individuals with recently acquired HCV infections or HCV reinfections is crucial. A reliable diagnostic tool, such as HCV RNA testing, is essential to expedite the diagnosis of HCV infection, given the delay of HCV antibody (anti-HCV antibody) seroconversion (12) and persistently positive anti-HCV antibody after primary HCV infection. However, HCV RNA testing by PCR assay is limited by the cost incurred in resource-limited regions, especially in the high-risk populations with risky exposures that require repeated testing. The HCV core antigen (HCVcAg) assay can be an alternative method to diagnose HCV infections at a lower cost than for HCV RNA testing. Moreover, HCVcAg can be detected 1.5 months (38 to 50 days) earlier than anti-HCV antibody and 1 to 2 days after the appearance of HCV RNA in the blood samples of individuals with recently acquired HCV infections (13). A systematic review and meta-analysis concluded that the HCVcAg assay had a high sensitivity and specificity, in which the HCVcAg levels correlated well with HCV RNA levels that were greater than 3,000 IU/mL (14).

To ensure scale-up of HCV testing and treatment without unnecessary delays and restrictions, efforts should be made to increase the access to and reduce the costs for HCV testing and treatment, particularly in resource-constrained settings. We have recently demonstrated the feasibility and cost reductions of regular (every 3 months) pooled-plasma HCV RNA testing to identify HCV infections among at-risk PLWH with sexually transmitted infections (STIs), elevated aminotransferases within the past 6 months, or past HCV infections (15). However, whether the HCVcAg assay could also play a role in this setting remains less investigated. Thus, the present study aimed to assess the performance of the HCVcAg assay in populations at risk for recently acquired HCV infections.

(The preliminary data of the present study have been presented in an abstract form [abstract no. 541] at the Conference on Retroviruses and Opportunistic Infections [CROI] 2022 [16].)

## RESULTS

**FIG 1:**
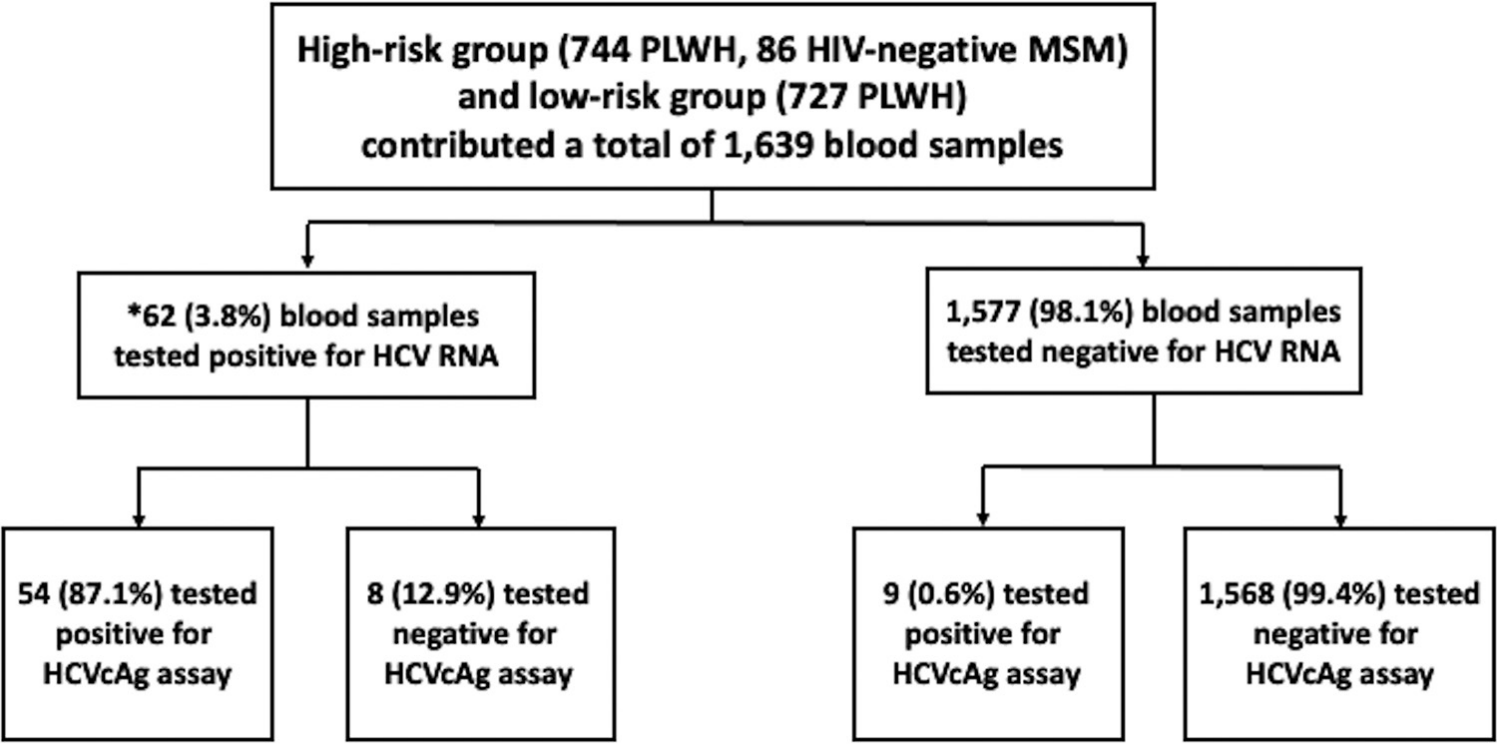
Study flow. *, among the 34 HCV-seronegative PLWH at enrollment in whom HCV viremia was subsequently detected by pooled-serum HCV RNA testing, anti-HCV antibody remained negative in 44.1% (15/34) at the time of detection of HCV viremia. HCVcAg, hepatitis C virus core antigen; MSM, men who have sex with men; PLWH, people living with HIV.

Between 25 June 2019 and 25 February 2021, 1,639 samples from 830 high-risk participants (744 PLWH and 86 HIV-negative participants) and 727 low-risk PLWH at enrollment were tested for HCVcAg, including 61 PLWH with 62 samples that tested positive for HCV RNA by pooled-plasma HCV RNA testing (Fig. 1). The demographic and clinical characteristics of these participants are shown in Table 1. HIV-negative participants were much younger than the other two groups (mean age of 28.7 versus 38.4 and 42.6 years for high- and low-risk PLWH, respectively; *P* < 0.001). Low-risk PLWH were more likely to be heterosexuals (11.1%) and have positive results for hepatitis B virus surface antigen (HBsAg) (12.4%), while high-risk PLWH and HIV-negative participants were mostly MSM (97.2% and 97.7%, respectively) and less likely to have positive HBsAg (8.2% and 3.6%, respectively). Anti-HCV antibody was positive at baseline in 27.4% of high-risk PLWH and 0% in the other two groups. All PLWH received combination antiretroviral therapy, with 96.4 to 98.8% having CD4 counts of ≥200 cells/mm^3^ and 94.1 to 99.4% having plasma HIV RNA loads of <50 copies/mL. Of 744 high-risk PLWH at enrollment, 76.5% had STIs, 30.4% had ever achieved a sustained virologic response (SVR) after 12 weeks off therapy (SVR12) or spontaneous clearance of HCV, and 6.5% had elevated aminotransferases. For 86 HIV-negative participants, 81.4% were receiving preexposure prophylaxis for HIV and 18.6% had STIs at enrollment.

**TABLE 1:** Characteristics of participants who underwent both HCV core antigen assay and pooled HCV RNA testing

Characteristic^*a*^	Value for participants who were:			*P* value^*c*^
	PLWH considered^*b*^:		HIV negative	
	High risk	Low risk		
No. of participants	744	727	86	
Age (yr) (mean [SD])	38.4 (8.7)	42.6 (11.2)	28.7 (5.6)	<0.001
Male sex (no. [%])	743 (99.9)	692 (95.2)	86 (100.0)	<0.001
Risk group for HIV transmission (no. [%])				
MSM	723 (97.2)	618 (85.0)	84 (97.7)	<0.001
Heterosexuals	10 (1.3)	81 (11.1)	2 (2.3)	
People who inject drugs	11 (1.5)	7 (1.0)	0 (0)	
Positive HBsAg (no. with result/no. tested [%])	(61/743) (8.2)	90/726 (12.4)	3/84 (3.6)	0.004
Anti-HCV antibody positive at baseline (no. with result/no. tested [%])	200/731 (27.4)	0 (0)	0 (0)	<0.001
CD4 count of >200 cells/mm^3^ (no. with result/no. tested [%])	692/718 (96.4)	715/724 (98.8)	NA^*d*^	NC^*e*^
PVL <50 copies/mL (no. with result/no. tested [%])	699/743 (94.1)	723/727 (99.4)	NA	NC
On ART (no. [%])	741 (100)	730 (100)		
Reason for inclusion in the study (no. [%])				
Having had sexually transmitted infections	569 (76.5)	NA	16 (18.6)	
Having achieved SVR12 or spontaneous clearance of HCV	226 (30.4)	NA	NA	
Elevated aminotransferases	48 (6.5)	NA	NA	
Preexposure prophylaxis for HIV (no. [%])	NA	NA	70 (81.4)	

a ART, antiretroviral therapy; HBsAg, hepatitis B virus surface antigen; HCV, hepatitis C virus; MSM, men who have sex with men; PVL, plasma HIV RNA load; SVR12, sustained virologic response after 12 weeks off therapy.

b PLWH, people living with HIV.

c *P* value is for comparison among the three groups.

d NA, not available.

e NC, not calculable.

All of the 62 samples that tested positive for HCV RNA by pooled-plasma HCV RNA testing were from 61 high-risk PLWH, including 30 PLWH testing positive at enrollment, 11 at week 12, 9 at week 24, 9 at week 36, 2 at week 48, and 1 at an unscheduled visit. Therefore, the overall rate of HCV viremia was 8.2% (61/744) in high-risk PLWH, 0% in HIV-negative participants, and 0% in low-risk PLWH. Of the 62 samples that tested positive for HCV RNA by pooled-plasma HCV RNA testing, 54 also tested positive for HCVcAg, leading to a sensitivity of the HCVcAg assay of 87.1% (54/62). Among 1,577 specimens that tested negative for HCV RNA by pooled-plasma HCV RNA testing, 1,568 also tested negative for HCVcAg, resulting in a specificity of the HCVcAg assay of 99.4% (1,568/1,577). Given the overall prevalence of HCV viremia of 3.8% (62/1,639) of all samples tested, the positive predictive value (PPV) of the HCVcAg assay was 85.7% (54/63) and the negative predictive value (NPV) was 99.5% (1,568/1,576). The accuracy of the HCVcAg assay was 98.96%.

**FIG 2:**
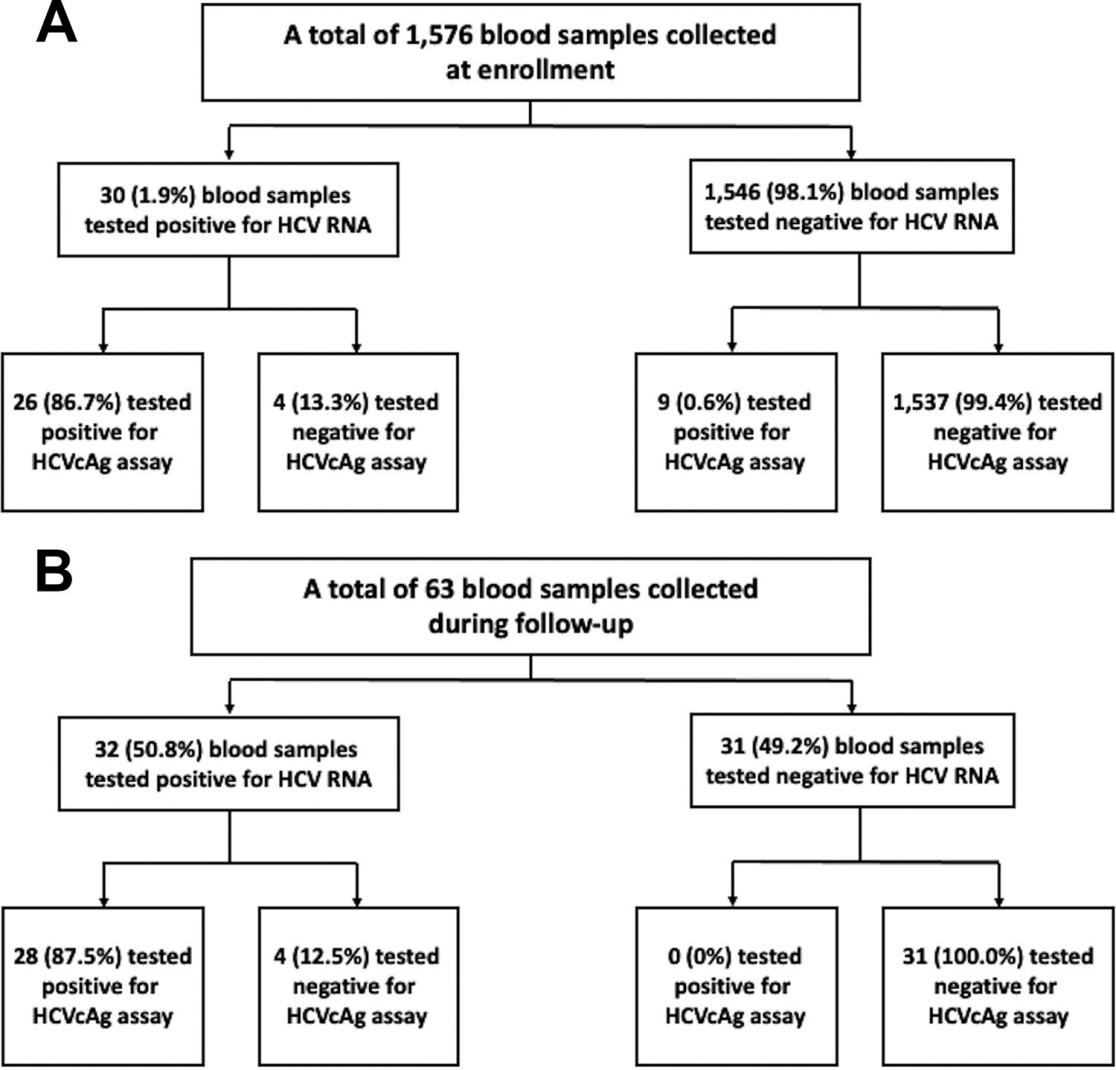
Performance of HCVcAg assay in blood samples collected at enrollment (A) and during follow-up (B). HCVcAg, hepatitis C virus core antigen.

Since the HCVcAg assay had good performance in the setting of established or chronic HCV infection, we grouped the blood samples into two groups, one collected at enrollment and the other during follow-up (Fig. 2A and B), and repeated the analyses of sensitivity, specificity, PPV, and NPV for the HCVcAg assay. For the 1,576 blood samples collected at enrollment, the sensitivity, specificity, PPV, NPV, and accuracy of the HCVcAg assay were 86.7%, 99.4%, 74.3%, 99.7%, and 99.2%, respectively (Fig. 2A). As for the 63 blood samples collected during follow-up, the sensitivity, specificity, PPV, NPV, and accuracy of HCVcAg assay were 87.5%, 100%, 100%, 88.6%, and 93.7%, respectively (Fig. 2B). The correlation between the performance of HCVcAg and the HCV genotype of the specimens with detectable HCV RNA is shown in Table 2. The correlation was stronger for HCV genotypes 2 and 6 than for genotype 1 and unidentified/indeterminate genotype.

**TABLE 2:** Correlation between the performance of the HCVcAg assay and HCV genotype

HCV genotype (no. of specimens with genotype)^*a*^	Pearson’s correlation (*r*)	*P* value
1 (24)	0.355	0.089
2 (13)	0.806	0.001
6 (22)	0.875	<0.001
Unidentified/indeterminate (3)	−0.199	0.873

a HCV genotype 1 included 1a in 12 specimens, 1b in 9, and 1 in 3.

To examine whether HIV and other viral hepatitis might have any impact on the performance of the HCVcAg assay, we repeated the analyses by categorizing the participants into three groups: (i) HIV-negative MSM testing negative for both HBsAg and anti-HCV antibody, (ii) PLWH testing positive for HBsAg, and (iii) PLWH testing negative for HBsAg. The results of the analyses are shown in Tables 3 to 5. Of the 86 HIV-negative MSM, 81 tested negative for both HBsAg and anti-HCV antibody. The sensitivity, specificity, PPV, and NPV of the HCVcAg assay in this group were not calculable, 98.8%, 0%, and 100%, respectively (Table 3).

**TABLE 3:** Performance of the HCVcAg assay in the participants who tested negative for both HBsAg and anti-HCV antibody among the HIV-negative MSM

HCVcAg result	No. of participants with indicated HCV RNA result^*a*^	
	Positive (*n* = 0)	Negative (*n* = 81)
Positive (*n* = 1)	0	1
Negative (*n* = 80)	0	80

a Analysis of the performance of the HCVcAg assay in the group comprising 81 participants who tested negative for both HBsAg and anti-HCV antibody among the 86 HIV-negative men who have sex with men (MSM). Sensitivity, not calculable; specificity, 98.77% (80/81); positive predictive value, 0% (0/1); negative predictive value, 100% (80/80); accuracy, not calculable.

**TABLE 4:** Performance of the HCVcAg assay in the participants who were HBsAg positive among the high-risk and low-risk PLWH

HCVcAg result	No. of participants with indicated HCV RNA result^*a*^	
	Positive (*n* = 4)	Negative (*n* = 153)
Positive (*n* = 8)	4	4
Negative (*n* = 149)	0	149

a Analysis of the performance of the HCVcAg assay in the group comprising 151 participants who were HBsAg positive and had 157 blood samples for analysis from the 744 high-risk and 727 low-risk people living with HIV (PLWH). Sensitivity 100.00% (4/4); specificity, 97.39% (149/153); positive predictive value, 50.00% (4/8); negative predictive value, 100.00% (149/149); accuracy, 97.45%.

**TABLE 5:** Performance of the HCVcAg assay in the participants who were HBsAg negative among the high-risk and low-risk PLWH

HCVcAg result	No. of participants with indicated HCV RNA result^*a*^	
	Positive (*n* = 58)	Negative (*n* = 1,319)
Positive (*n* = 54)	54	0
Negative (*n* = 1,323)	4	1,319

a Analysis of the performance of the HCVcAg assay in the group comprising 1,318 participants who tested negative for HBsAg and had 1,377 blood samples for analysis from the 744 high-risk and 727 low-risk people living with HIV (PLWH). Sensitivity, 93.10% (54/58); specificity, 100% (1,319/1,319); positive predictive value, 100% (54/54); negative predictive value, 99.70% (1,319/1,323); accuracy, 99.71%.

The respective HCV RNA loads of the 8 blood samples that tested positive for HCV RNA but negative for HCVcAg were 305, 429, 880, 1,435, 1,745, 4,560, 6,600, and 7,150 IU/mL. The mean HCV RNA load of 54 samples with concordantly positive results between pooled-plasma HCV RNA testing and the HCVcAg assay was 6.2 (range, 1.3 to 8.5) log_10_ IU/mL, which was significantly higher than that (3.2 [range, 2.5 to 3.9] log_10_ IU/mL) of the 8 samples with discordant results between pooled-plasma HCV RNA testing and the HCVcAg assay (*P* < 0.001) (Table 6). Among the 62 samples that tested positive for HCV RNA by pooled-plasma HCV RNA testing, 55 (88.7%) had HCV RNA loads of 3,000 IU/mL or greater and 7 (11.3%) had HCV RNA loads of less than 3,000 IU/mL. Of those with HCV RNA loads of 3,000 IU/mL or greater, 94.5% (52/55) tested positive for HCVcAg, while only 28.6% (2/7) tested positive for HCVcAg among those with HCV RNA loads of less than 3,000 IU/mL (*P < *0.001) (Table 7).

**TABLE 6:** Comparison of plasma HCV RNA loads between samples with positive HCV RNA but negative HCVcAg and samples with concordantly positive results of the two tests

Variable^*a*^	No. (%) of samples	Mean (range) HCV RNA load (log_10_ IU/mL)
HCV rNA positive/HCVcAg negative	8 (12.9)	3.2 (2.5–3.9)
HCV rNA positive/HCVcAg positive	54 (87.1)	6.2 (1.3–8.5)
*P* value		<0.001

a HCVcAg, hepatitis C virus core antigen.

**TABLE 7:** Comparison of the proportions of positive HCVcAg results between blood samples with lower and higher HCV RNA loads

HCV RNA load	No. (%) of samples	No. (%) positive for HCVcAg^*a*^
<3,000 IU/mL	7 (11.3)	2 (28.6)
≥3,000 IU/mL	55 (88.7)	52 (94.5)
*P* value		<0.001

a HCVcAg, hepatitis C virus core antigen.

## DISCUSSION

Our study showed that the sensitivity and specificity of the HCVcAg assay were 87.1% and 99.4%, respectively, in the diagnosis of recently acquired HCV infections among high-risk PLWH who had an overall prevalence of HCV viremia of 8.2%. In line with the previous report that positivity for HCVcAg correlated well with HCV RNA levels greater than 3,000 IU/mL (14), we also found that blood samples with HCV RNA loads equal to or greater than 3,000 IU/mL were more likely to test positive for HCVcAg than those with HCV RNA loads of less than 3,000 IU/mL (94.5% versus 28.6%; *P < *0.001). Likewise, the mean HCV RNA load of the samples that tested positive for both HCV RNA and HCVcAg was significantly higher than that of the samples that tested positive for HCV RNA but negative for HCVcAg (6.2 versus 3.2 log_10_ IU/mL; *P < *0.001).

The application of the HCVcAg assay has been assessed in the diagnosis of chronic HCV infection, monitoring of DAA treatment response, and cost-effect analysis in HIV-uninfected people and PLWH (17, 18). In contrast, our study was designed to investigate the performance of HCVcAg in the diagnosis of recently acquired HCV infections in high-risk populations, including PLWH and HIV-uninfected people taking preexposure prophylaxis (PrEP) for HIV. The results of these studies in individuals with chronic HCV infection showed that the HCVcAg assay was a cost-saving tool to diagnose HCV viremia and document treatment adherence but did not work well to determine SVR. While the sensitivity of the HCVcAg assay was lower in the setting of plasma HCV RNA loads of less than 3,000 IU/mL in this study and the study by Freiman et al. (14), often referred to as very low viral loads (VLVLs), the prevalence of VLVLs has been shown to be low (5.3%) among HCV treatment-naive persons, and 33% of blood samples with VLVLs tested positive by the HCVcAg assay in the Swiss Hepatitis C Cohort Study (19). Furthermore, patients with VLVLs might have a favorable disease course, with persistent VLVLs in 14% and spontaneous viral clearance in 17% (19). Therefore, in a setting of limited resources, such a missed proportion of HCV-infected patients with VLVLs by HCVcAg assay at a reasonable cost might be acceptable, though the impact of individuals with undiagnosed HCV infection who have VLVLs on the program aiming to achieve HCV elimination warrants further investigations.

With the rapid spread of HCV infection mainly among PLWH who are MSM worldwide in the past 2 decades (20), the HCVcAg assay has also been evaluated as an alternative HCV screening tool to identify new HCV infections among PLWH (21, 22) or for PLWH with acute HCV infections (23). Despite the compromised performance in the specimens with lower plasma HCV RNA loads, the HCVcAg assay could also be a cost-saving diagnostic tool for recently acquired HCV infections, given the high HCV RNA loads in the setting of recently acquired HCV infections, with a mean plasma HCV RNA load of 6.4 log_10_ IU/mL (15, 24), especially in the high-risk populations requiring repeat testing. Among PLWH with acute HCV infection, the sensitivity of the HCVcAg assay ranges from 89% to 100% and its specificity from 95% to 100% (21–23). To improve its sensitivity, confirmation by HCV RNA testing for a detectable signal of HCVcAg assay without restriction by its cutoff ratio of 3.00 fmol/L (23) or the addition of alanine aminotransferase to the current screening methods (22) was suggested. Likewise, our findings also support the HCVcAg assay as an alternative method to detect patients with recently acquired HCV infection, although its sensitivity remains a concern. Furthermore, the performance of the HCVcAg assay in blood samples collected at enrollment, assumed to consist of individuals with established or chronic HCV infection, and during follow-up, assumed to consist of individuals with acute or recently acquired HCV infection, was similar (Fig. 2).

Several limitations deserve to be acknowledged. First, the detection limit of pooled HCV RNA testing increased from 15 IU/mL by individual HCV RNA testing to approximately 300 IU/mL, which might lead to compromised sensitivity. Nevertheless, our sensitivity analysis confirmed that the results of pooled-plasma HCV RNA testing were the same as the ones by individual HCV RNA testing. Second, our study tested blood samples collected every 3 to 6 months; therefore, we were not able to compare the exact timing when HCV RNA or HCVcAg could first be detected in the incident cases of acute HCV infections that occurred between the two time points of blood sampling. Third, we did not perform a cost-benefit analysis of the implementation of HCVcAg to identify populations with different risks for HCV infection, which can inform testing policy in the test-and-treat strategy for HCV. Further assessments of the turnaround time required and direct and indirect costs incurred between pooled-plasma HCV RNA testing and individual HCVcAg testing are warranted in different epidemiologic and clinical settings. In our study, it took about 9 h to go through stages 1 to 3 (approximately 2.7 h for each stage; see Materials and Methods) for pooled-plasma HCV RNA testing to get the results and 30 min for the HCVcAg assay (15). Fourth, since the prevalence of a disease of interest will have an impact on the performance of an assay, our findings can only be applied to settings with an overall prevalence of HCV viremia of 3.8% (62/1,639 in our study) of all samples tested and may not be generalizable to other populations with different prevalences of HCV viremia. Fifth and last, the risk stratification for HCV infection in the participants might change with time, as one HIV-positive participant in this study belonged to the low-risk group initially but was later classified in the high-risk group during follow-up after he acquired syphilis (data not shown), which argues for the need for regular HCV testing among at-risk populations.

We conclude that the HCVcAg assay has high specificity and NPV in the diagnosis of recently acquired HCV viremic infection among PLWH and HIV-negative MSM at risk for HCV infection. However, its sensitivity and PPV are compromised in those with low HCV RNA loads.

## MATERIALS AND METHODS

### Study setting and population.

Increasing trends of recently acquired HCV infections had been noted among PLWH who were predominantly MSM and HCV seronegative at baseline in Taiwan during 1994 to 2018, with an HCV seroincidence rate reaching 28.10 per 1,000 person-years of follow-up (PYFU) in 2018 (25, 26). The rate of HCV reinfection was estimated to be 82 per 1,000 PYFU (27, 28). Syphilis was consistently found to be statistically significantly associated with HCV infections in all these studies (25–28). In Taiwan, DAAs have been reimbursed by the National Health Insurance (NHI) since 2017 (29, 30). Hepatologists and HIV-treating physicians are responsible for HCV care and treatment among eligible PLWH. The program had not reimbursed DAA retreatment for those who had ever received NHI-reimbursed DAAs until 2021, when a second course of DAAs could be reimbursed for those who had recurrent HCV infections due to relapse or reinfection. Enrollment in the HCV treatment program with achievement of SVR was high among PLWH (31–33).

In this study, we included populations at high risk for acquiring HCV infections, which included PLWH with STIs, elevated aminotransferases within the past 6 months, or having achieved spontaneous HCV clearance or sustained virologic response (SVR) by antivirals, and HIV-negative MSM with STIs or on preexposure prophylaxis (PrEP) for HIV. Individuals with previously documented HCV viremia but remaining untreated were excluded. The low-risk populations included were PLWH who were negative for anti-HCV antibody at baseline and without STIs or elevated aminotransferases in the past 6 months. The high-risk group underwent pooled-plasma HCV RNA testing every 12 weeks or when clinically indicated, and the low-risk group underwent this testing every 24 weeks (15). Participants were followed until the detection of HCV RNA, loss to follow-up, death, or completion of the 48-week follow-up. Participants with newly identified HCV viremia were linked to NHI-reimbursed DAA treatment if eligible. Repeated participation in the pooled-plasma HCV RNA testing was allowed for those testing negative for HCV RNA who had recurrent STIs or elevated aminotransferases after completing the 48-week follow-up or those having tested positive for HCV RNA who had achieved viral clearance either spontaneously or with DAA treatments.

### Laboratory investigations.

Given the estimated incidence of recently acquired HCV infection of 3.4 to 8.4% (25, 26), 3-stage pooled-plasma HCV RNA testing that combined 20 individual samples (50 microliters from each individual sample) into a pooled sample for HCV RNA testing (stage 1) was found to be the most cost-effective strategy in simulation with the size of 10,000 specimens based on the prevalence of HCV viremia of 1.8% (15). All of the 20 individual samples would be considered free of HCV if the pooled sample tested negative for HCV RNA. For any positive pooled samples, every 5 (150 microliters from each individual sample) of the 20 individual samples were randomly selected and combined into a mini-pooled sample for testing (stage 2). For a positive mini-pooled sample, each of the 5 samples was retested individually to identify the one(s) with HCV RNA (stage 3) (15).

The samples from PLWH and HIV-negative participants at high risk for HCV infection and low-risk PLWH at enrollment and all of the archived samples preceding the detection of HCV RNA during follow-up were tested for HCVcAg individually. This prospective study was approved by the Research Ethics Committee of the hospital (registration number 201904086RIPB), and all participants gave written informed consent.

HCV RNA was determined using the COBAS AmpliPrep HCV test (version 2.0; Roche, USA) before and the cobas 6800 (Roche, USA) after September 2020 (detection limit of 15 IU/mL), HCV core antigen was determined using the Abbott Architect HCV Ag assay (Abbott, Germany) (34), and anti-HCV antibody was determined using a fourth-generation enzyme immunoassay (Dia.Pro Diagnostic Bioprobes Srl, Italy) (26). The detection limit of the stage 1 pooled sample was approximately 300 IU/mL after pooling of 20 individual samples. We did a sensitivity analysis to compare the detection rates by individual and pooled testing for the 107 stage 1 pools (consisting of a total of 2,140 individual samples) (15), and all the results of the pooled-plasma HCV RNA testing were the same as those of individual HCV RNA testing. Thus, pooled-plasma HCV RNA testing was used as a reference for HCV viremia in the current study. Anti-HCV antibody in the specimens with detectable HCV RNA was determined individually if the participants had negative anti-HCV antibody before they tested positive by pooled-plasma HCV RNA testing. The cost of individual HCV RNA testing is 2.5 times higher than that of individual HCVcAg testing; however, the cost for identifying samples with HCV RNA could be reduced by 85% with the use of pooled-plasma HCV RNA testing (15).

### Statistical analysis.

Categorical variables were compared using the *X*^2^ or Fisher’s exact test, and noncategorical variables using Student’s *t* test or the Mann-Whitney *U* test. The correlation between HCV RNA loads and HCVcAg was measured using Pearson’s correlation coefficient in the Statistical Program for Social Sciences (SPSS Statistics version 21; IBM Corp., Armonk, New York).

### Data availability.

Deidentified participant-level data will be available on publication of the study. Requests for data should be sent to Ming-Lung Yu at the e-mail address in the corresponding author footnote and, on review of the proposed protocol and signing of a data sharing agreement, the data will be made available. The protocol and consent form will also be available upon email request.
